# Perceptions of a group of hospital pharmacists and other professionals of the implementation of clinical pharmacy at a high complexity public hospital in Brazil

**DOI:** 10.1186/s12913-018-3036-7

**Published:** 2018-04-04

**Authors:** Thaciana dos S. Alcântara, Thelma Onozato, Fernando de C. Araújo Neto, Aline S. Dosea, Luiza C. Cunha, Dyego C. S. A. de Araújo, Déborah Pimentel, Divaldo P. Lyra Junior

**Affiliations:** 1 Education and Research Laboratory of Social Pharmacy, Federal University of Sergipe, São Cristóvão, Sergipe Brazil; 2 Federal University of Sergipe, Avenida Marechal Rondon, Jardim Rosa Elze, São Cristóvão, Sergipe Brazil

**Keywords:** Hospital, Clinical pharmacy, Implementation of health services, Perceptions of health professionals

## Abstract

**Background:**

During the process of implementation of clinical pharmacy services, internal and external factors may favor or hinder the incorporation of care into the hospital routine. This study aimed to understand the perceptions of a group of hospital pharmacists and other professionals of the implementation of clinical pharmacy at a high complexity public hospital in Brazil.

**Methods:**

A focus group with 16 pharmacists and interviews with tree key stakeholders including managers in the pharmaceutical, medical, and nursing profession were conducted to understand their perceptions of the implementation clinical pharmacy services in a high complexity public hospital in Brazil. The service proposal was presented to the selected participants before conducting the focus group. Professionals with an overview of the hospital and influence on the relevant departments for the implementation of clinical pharmacy at the institution were selected. Data collected were transcribed and analyzed using the Bardin Content Analysis technique. Data analyzed were systematized into categories and registration units. The methodology involves the organization and analysis of reported content to make inferences.

**Results:**

The data obtained were divided into four categories: “Perception of the current situation”, “Implementation expectations”, “Barriers to implementation”, “Implementation facilitators”. Participants discussed the stagnation of clinical activities of the pharmaceutical profession in Brazil, a reality that results from a lack of clinical training in the country. Pharmacists expressed their expectations for changes in professional performance. According to the managers, such services would positively affect clinical outcomes for patients. Gaps in academic education, lack of knowledge, and poor communication skills were barriers reported in this study. Pharmacists’ clinical experience has been reported to facilitate the provision of services.

**Conclusions:**

This study highlights factors that may influence the implementation of clinical pharmacy services in the institution analyzed, such as resistance, fear, and frustration as barriers, as well the experience in clinical pharmacy of some pharmacists in the institution was one of the facilitators most cited by participants. This knowledge may aid future planning for the implementation of clinical pharmacy in hospitals.

## Background

Recent studies have reported that the inclusion of clinical pharmacy services can minimize medication errors, reduce hospital costs, and improve pharmacotherapy outcomes for patients. Perez et al. (2009), for example, showed that clinical pharmacy was of benefit in 69% of the studies analyzed, and the average reduction in hospital costs was USD 4.81 for each dollar spent. In the study by Rivkin and Hongjun (2011), it was observed that involvement of the clinical pharmacist during hospital rounds reduced the number of drug interactions in intensive care patients by 65%. A more recent study showed that the clinical pharmacy service prevented 64.19% of medical errors in a group of hospitalized patients with diabetes, 18 times more than the control group, saving the hospital USD 191.08 within 15 days [1–4].

Because of the evidence proving the benefits of clinical pharmacy services, such as medication reconciliation, prescription analysis, pharmacotherapy follow-up, and pharmacotherapy review, the World Health Organization (WHO) promotes the provision of these services in every country. Despite the regulation of clinical pharmaceutical duties in Brazil by means of the resolution of the Federal Council of Pharmacy (CFF) in 585/2013, this is still not an established practice [5–7].

In fact, pharmaceutical education in developing countries, as well as in countries in Europe, are still developed in a manner disjointed from social reality [8–10]. Academic education is focused on the production of drugs, techniques, and tests. The health of the population occupies little space in the educational process, with few interdisciplinary activities and little interaction between academia and services [11–13]. Therefore, it is necessary to identify barriers and facilitators in the implementation of innovative practices in pharmaceutical services. Insight into the perceptions of the professionals involved is vital in improving the quality of these activities [14, 15].

In this context, the use of qualitative research has identified factors interfering with the structure, work processes, and professional skills needed to obtain positive results when implementing clinical pharmacy services [16–18]. According to Penm et al. major barriers to the consolidation of these services in developing countries include the shortage of qualified pharmacists and the lack of adequate infrastructure [19]. On the other hand, facilitators observed included patient satisfaction with the services and research support for the improvement of pharmaceutical practice [19–21].

In Brazil, this practice is still new, and there is a lack of data on the perspectives of pharmacists and other professionals involved in implementing clinical pharmacy services in hospitals. Thus, this study aimed to understand the perception, to explain the relationships, values, attitudes, beliefs, and habits of a group of pharmacists and managers representing pharmacists, doctors, and nurses immersed in experiences preceding the creation and implementation of clinical pharmacy services in a high complexity public hospital.

## Methods

A qualitative study was conducted using a focus group and interviews with key stakeholders. The study methodology involved objectively collecting data on the understanding and interpretation of phenomena in order to understand the perceptions of the individual [22, 23].

The study was conducted in pharmacy units of a high complexity public hospital, the Emergency Hospital of Sergipe (HUSE), located in the northeast of Brazil. HUSE is the largest public hospital and main facility of the Unified Health System (SUS) for complex cases in Sergipe. With an average attendance of 14,000 patients per month in the Urgent/Emergency sectors alone, HUSE comprises 13 hospital wards and has a capacity of 421 beds. The hospital has 7 pharmacy units located in pediatrics, oncology, intensive care, emergency room, center, and surgical center.

### Study context

Currently, pharmacists carry out only logistic activities in pharmacies. These activities, which are considered a priority in hospital pharmacy, include cost-effective planning, implementation, and control, of the flow and storage of medical supplies, drugs, and other materials. However, clinical activities with the application of knowledge of drug functions and interactions are not carried out in the pharmacy.

In view of this reality, by reason of mutual interest, a partnership between the Education and Research Laboratory of Social Pharmacy of the Federal University of Sergipe and the hospital institution was established. This partnership acts as an advisory and training body for pharmacists and employees of pharmacies, with the aim of promoting clinical pharmacy services (e.g. drug conciliation, prescription analysis, pharmacotherapy follow-up, and pharmacotherapy review) in HUSE pharmacy units.

In 2016, the study was in the phase of diagnostic analysis to evaluate both the group’s expectations and the need to implement clinical services at this institution. This evaluation directed the training sessions on communication skills, interpretation of laboratory tests, drug administration, and clinical pharmacy services were carried out.

### Focus group and interviews

All 28 hospital pharmacists of the institution were invited to participate in the study. The service proposal was presented to the selected participants before conducting the focus group. Key stakeholders including the managers of pharmaceutical, medical, and nursing departments were interviewed. Professionals with an overview of the hospital and influence on the relevant departments crucial for the implementation of clinical pharmacy at the institution were selected. These individuals were also likely to be responsible for organizing the working hours of the professionals, leadership, and group representatives. Therefore, we interviewed the hospital pharmacy manager as pharmaceutical managers; the epidemiological surveillance doctor as a medical manager; and the risk management nurse as a nursing manager. Moreover, the same questions were posed for the focus group and interviews. Data saturation was reached when there was enough information to replicate the study, when the ability to obtain additional new information has been attained, and when further coding was no longer feasible.

Following the recommendations of the consolidated criteria for reporting qualitative research (COREQ) [24], the focus group and interviews were conducted on different days. First, the focus group, and then interviews, were carried out in a separate environment from the workplace to minimize potential interference. All participants signed a consent form, which authorized video recording and publication of data from the focus group and interviews. In addition, this study was approved by the Ethics Committee of the Federal University of Sergipe (CAAE number: 36927014.4.0000.5546).

### Interview guide development

Prepared by the project researchers, the interview guide for each discussion (both focus group and interviews) probed the perception of pharmacists and other professionals regarding the institution where they work, knowledge of clinical pharmacy services, expectations regarding implementation, and barriers they expect to face in implementing the services (Annex).

The interview guide was composed of questions open-ended (enables the respondent responds freely to what he thinks about the subject), conducted by a moderator (a researcher) who maintained a neutral relationship with study participants, created a stimulating environment for the exchange of views, and maintained the focus of discussion. The focus group and interviews was conducted in Portuguese, lasted a maximum of 2 h; the entire discussion was recorded on video and/or voice recorded and was later transcribed for data analysis.

### Analysis

Data collected were transcribed and analyzed using the Bardin Content Analysis technique [25]. Data were organized and systematized into categories and registration units. The methodology involves the organization and analysis of reported content to make inferences. This technique consists of three stages: 1) pre-analysis, in which the material to be analyzed is organized, the initial ideas are systematized, and text clippings are created in document analysis; 2) exploration of the material, in which data are grouped into categories. The categories were previously determined, derived from the questions made to the interviewees and after the interviews were organize according to the similarity of the subject; 3) interpretation, where the material is interpreted and inferences are made. Two researchers (TSA and TO) independently analyzed the focus group discussions and interviews; in case of disagreement, a third investigator (ASD) was requested. Finally, critical analysis of the categories of the focus group and interviews was conducted.

## Results

Of the 28 pharmacists invited to participate in the focus group, 16 pharmacists accepted to participate in the focus group (Table 1). Of the 12 pharmacists who refused to participate, 10 cited lack of time and 2 did not view the e-mail invitation to the focus group. No manager refused to participate in the interviews. The recording of interviews and the focus group produced a total of 73 and 101 min of audio, respectively, fully transcribed for analysis. First the focus group, then the interviews were analyzed. The data obtained from both had the same analysis criteria and were divided into four categories: “Perception of the current situation”, “Implementation expectations”, “Barriers to implementation”, “Implementation facilitators” (Table 2).

**Table 1:** Characteristics pharmacists (*n* = 13) and managers (*n* = 3)

Pharmacists		
Characteristic	No. (%)	
Sex		
Male	04 (25)	
Female	12 (75)	
Postgraduate degree		
Yes	03 (19,75)	
No	13 (81,25)	
	Average	Standard deviation
Age, yr	34,3	5,28
Time of graduation, yr	11,8	4,51
Working time in the institution, yr	5,18	4,94
Managers		
Characteristic	No. (%)	
Sex		
Male	01 (33,33)	
Female	02 (66,66)	
Postgraduate degree		
Yes	3 (100)	
No	0 (0)	
	average	Standard deviation
Age, yr	37,6	10,69
Time of graduation, yr	15	11,26
Working time in the institution, yr	5,3	2,08

**Table 2:** Perception of pharmacists and managers regarding the implementation of Clinical Pharmacy services in a high-complexity public hospital. Categories of “Perception of the current situation”, “Expectations of clinical pharmacy implementation”, “Barriers to the implementation” and “Facilitators for the implementation”. Aracaju, 2016

Category	Registration Unit	
Perception of the current situation	Pharmacists	Managers
	Profession in Brazil does not renovate / it is outdated Dissatisfaction with work Exclusively distributive assignments Job satisfaction when there is interprofessional collaboration	Incipient pharmaceutical education in the clinical area Hospital without work process focused on patient safety There is no record of clinical interventions carried out by the pharmacists Lack of support from pharmacy teams to clinical services Fragmented view of the medical staff on patients Lack of clinical pharmacy services in the hospital Some pharmacists of the team prefer distributive assignments Management support to activities related to patient safety
Expectations	Pharmacists	Managers
	Professional practice focusing on clinical services Researchers’ support and guidance Positive impact of the services for patients and healthcare team Services sustainability The new services will make pharmacists more visible for directors	Improvement in the medication use process Positive impact for patients Reducing costs and damage caused by misuse of drugs Improvement of the communication process among the healthcare team Appreciation of pharmacist’s clinical performance at the hospital Pharmacist’s qualification Recruitment of new pharmacists
Barriers	Pharmacists	Managers
Subcategory External factors	Failures of the public health system in Brazil Resistance of some healthcare professionals to clinical pharmacy services Lack of proactive attitude and collaboration among pharmacists	Changes in the hospital board of directors may hinder the process of Clinical Pharmacy implementation Unawareness of pharmacists’ clinical activities by healthcare team and managers Resistance of some healthcare professionals to clinical pharmacy services
Structure and organization	Insufficient pharmacists to distributive and clinical tasks Lack of adequate working facilities to develop clinical pharmacy Drug shortage in the hospital Manager pressure on pharmaceutical distributive tasks Poor workforce management Lack of standardization of pharmaceutical work process	Insufficient number of pharmacists and pharmacy assistants to assist the hospital’s patients Pharmacists have to deal with clinical and distributive tasks High rotation of physicians in the hospital Lack of standardization of distributive activities Lack of standardization of pharmaceutical work process Lack of integration (collaborative practices) in the healthcare team work processes
Barriers	Pharmacists	Managers
Subcategory Structure and organization	Lack of training of pharmacy assistants on distributive assignments Lack of experience and training of pharmacists on clinical practice	
Attitudes of pharmacists and relationship with healthcare team	Pharmacist’s insecurity by poor qualification and experience on clinical practice Lack of pharmacist autonomy at work Difficulties in interacting with the healthcare team Unawareness of pharmacists’ clinical activities by healthcare team and managers	Uncertainty regarding the sustainability of Clinical Pharmacy services Absence of communication with the healthcare team
Facilitators	Pharmacists	Managers
Subcategory Structure and organization	Lack of training of pharmacy assistants on distributive assignments Lack of experience and training of pharmacists on clinical practice	
	Part of the pharmacist’s team has experience in Clinical Pharmacy Prior knowledge of other healthcare professionals on clinical pharmacy services Support of medical residents Existence of a Drug Information Service at the hospital	Part of the pharmacists’ team has clinical experience Interest of the majority of pharmacists to provide clinical services Good relationship between pharmacists and some healthcare professionals Management support to activities related to patient safety program Support of pharmaceutical and medical residents Organization of the healthcare team work processes Support of healthcare team, managers and pharmacy team Training of pharmacists and pharmacy assistants for clinical practice Adequacy of the physical structure for service Pharmacist participation in the multidisciplinary activities of the hospital Registration of clinical and economic impacts of the Clinical Pharmacy services

### Perception of the current situation

Perceptions regarding the pharmaceutical profession and the activities carried out by pharmacists were reported, both by the pharmacists themselves and by the managers of pharmacists, doctors and nurses. The pharmacists discussed the logistical nature of their duties and the stagnation of the profession’s clinical activities in Brazil. According to the managers, this situation is due to a lack of clinical education in the country, leaving pharmacists with inadequate clinical abilities to meet hospital demand. Some pharmacists reported this to generate dissatisfaction with work in the institution. *“We are only logistic pharmacists, working simply seeing stock, if there are missing or left over medicines, if ten or five vials are enough,” said a pharmacist.*The managers reported that the institution’s directors support the activities directed at the patient safety program. These activities require more interaction with the multidisciplinary team, and according to the pharmacists, there is professional satisfaction when this happens. However, such activities are not reality this hospital. As one manager said *“The hospital administration is supporting the Patient Safety Program, I guess they understand that the Clinical pharmacy is an important point on this road.”*

### Implementation expectations

Pharmacists reported having expectations for professional practice to focus on clinical practice, and according to the managers, this would allow improvement in the rational drug use process, reducing costs and damage caused by misuse of medicines in the hospital. Another expectation reported was in relation to the technical support provided by the researchers to develop clinical and communication skills, as well as acquiring knowledge on clinical pharmacy, as seen in the following statement: *“The expectation is that you (researchers) give us the direction, because as you realized that we do not have that background, I think that the priority would really be planning, such as step by step, what we will do first, what we will do after...” said a pharmacist.*

According to the managers, the development of clinical and communication skills, as well as the implementation of the services, may increase the qualifications and professional appreciation of pharmacists. Moreover, according to the pharmacists, they may become more visible to the hospital in providing these services. According to the managers, strengthening and support of these services may subsidize the hiring of new workers .

### Barriers to implementation

Issues that affect the execution of clinical pharmacy services in the hospital have been identified: external factors beyond the control of pharmacy, structural and organizational barriers, and barriers with respect to the attitudes of pharmacists and the relationship between the health team.

#### External factors

External factors identified by pharmacists related to lack of support from some hospital pharmacists, and according to one of the managers, resistance of the health professionals to new services being implemented in the hospital, acording another manager said: *“Here at the hospital the team members are resistant to novelties, so I think this will be the major barrier...”*

Managers reported the lack of pharmaceutical clinical activities by other health professionals to be a barrier. Flaws in the public health system (such as problems in the administration of public hospitals due to political issues), as well as lack of recognition of the importance of the pharmacist by the hospital management were also cited by pharmacists as barriers. Those involved in the project may have difficulties in overcoming these barriers, thus hindering the process of implementing the services. One pharmacist said: “Those who are there for more time know that the difficulties are huge, from the management... mismanagement, inefficiencies, problems in public service management.” Another pharmacist also commented: “The main barrier is the hospital management. It is necessary to make the management understand that the pharmacist is important for the hospital to start developing something, from there we will get structure, we will get everything.”

#### Structural and organizational

Participants reported barriers related to lack of time due to insufficient human resources and high workload (mainly bureaucratic, according to one of the managers). Moreover, the lack of adequate working facilities has often been reported, according a pharmacist said *“There are problems mainly in the physical structure, like furniture and technology.”.* Another obstacle is related to the physical structure (distance of pharmacies) of the hospital preventing communication with the health care team *“Few professionals in the hospital know where pharmacies are located ... maybe they are not in a suitable and strategic location, to generate demand for pharmaceutical services”*, said another pharmacist.

Lack of standardization of work processes of pharmacists and the health team was identified as an organizational problem. Another issue highlighted was the rigidity of the organizational structure, which did not promote autonomy and innovation, but focused the role of pharmacists on logistical activities. “The number of professionals for clinical pharmacy is small ... will it be possible to have clinical pharmacists without harm to the logistics area?”, said a manager.

#### Attitudes of pharmacists and relationship with the health team

Discussions pointed to lack of knowledge and skills among pharmacists, who feel insecure in providing clinical services at the hospital. Overall, pharmacists and managers reported the need to develop specific communication skills with patients and the health team. As a result, pharmacists reported feelings of shame, fear and frustration. The lack of communication with other health professionals was cited as a barrier by pharmacists. According to the managers’ report, this barrier may be related to lack of knowledge by the other health professionals having pharmaceutical clinical duties. As reported by doctors and nurses, fears that the pharmacist may start supervising their work may contribute to the lack of communication. According some pharmacists, *“The lack of qualification to perform this type of service is a barrier ... we do not have qualification for entering the health team ... we will have a hard time*” and *“Because we are faced with the difficulty of communication with the doctor and other professionals.”*

### Implementation facilitators

In this study, managers reported the support of the hospital board in the implementation of clinical pharmacy, as this service may contribute to patient safety program improvements.

Pharmacists stressed that clinical experience of some colleagues and prior knowledge of other health professionals within pharmaceutical clinical services favor the implementation process.

The organization of work processes of the health care team professionals, as well as participation by pharmacists in the multidisciplinary activities of the hospital were reported to facilitate project success. In addition, the adequacy of the hospital structure, continuous training, and recording of the clinical and economic impact of services have also been reported as facilitators. According a manager, “... it is the time of the pharmacist to take part of what's going on at the hospital, to debate solutions. The implementation of clinical pharmacy is integrated in the plans of the safe use of medicine. So, the participation of the pharmacist in these multidisciplinary meetings is essential for the other health team members to recognize their importance”.

Another point stressed by managers was the importance of both maintaining records and disclosing positive clinical and economic impacts of the clinical pharmacy services implemented. *“We must learn to make records of clinical and economic outcomes, statements and studies to demonstrate the benefits obtained, especially in the public sector...”* said a manager.

## Discussion

### Perception of the current situation

Pharmacists reported that their current assignments are mainly logistical. This generates dissatisfaction, as some of their clinical activities are not recognized by other health team professionals, perceptions also reported in other studies [26, 27]. According to Bermond et al. the pharmacist still takes predominantly administrative actions at the expense of health education actions such as guidance and promotion of responsible use of medicines [28]. With the training of health professionals based on the biomedical paradigm does not complement the pharmacists with the clinical skills [9, 29].

This can result in dissatisfaction and lack of appreciation of pharmacists by patients and the health team, as has been reported in some studies [30–32]. This dissatisfaction at work can be correlated with other negative effects such as absenteeism, work fatigue, burnout syndrome, poor quality of work, and reduced commitment to the profession. Any of these consequences is expensive and, moreover, may affect the way that other staff members and patients see the pharmacist and can therefore limit interactions with him [30, 33].

The support by the management of the institution of the activities related to the national patient safety program, reported in the study, may contribute to increasing satisfaction of these professionals, because this program includes clinical activities developed by the pharmacist along with the health team [34, 35]. According to Brandão et al. the patient safety programs are well defined, and represent a reality within the main Brazilian hospitals [36]. This program is linked to the quality programs of the Brazilian federal government. Its specific goals are to prevent avoidable damage and to minimize risks of incidents through the correct identification of patients, reduce hospital infections, and to reduce errors in surgery and medication. These are among the so-called nine solutions to patient safety, according to WHO [37]. In this context, clinical pharmacy is one of the strategic requirements for the quality of the health system, and one of the activities aimed at promoting patient safety [34, 37, 38].

### Implementation expectations

The expectations of participants were linked to an idealization of clinical pharmacy and the benefits that these services can bring to the institution. Some studies have identified the “drivers” of change in pharmacy practice that strengthens pharmacists’ expectations; for example, the desire for job satisfaction and to provide health care directly to the patient [39–42]. These expectations are strengthened by increasing the development and positive results of clinical pharmacy services, as shown in several countries [6, 43–45]. Moreover, reduced costs to the health system due to the rational use of medication stimulates interest in the development of these services [46, 47].

Despite the positive results for clinical pharmacy services in many countries, including Brazil, pharmacists have difficulty in performing clinical activities owing to lack of clinical and communication skills [19, 48, 49]. Thus, to provide these services, technical support is required during their establishment and implementation, with continuing education through training to develop knowledge and clinical skills [50]. In the study by Dosea et al. during the implementation of clinical services, pharmacists also reported expectations regarding technical support to improve their practices; this was important for the successful implementation of these services [20].

### Barriers to implementation

Barriers identified in this study, such as resistance to change, lack of time due to excessive bureaucratic and administrative burden, difficulty communicating with other professionals, and problems in the structure, have also been reported in similar studies conducted in hospitals and other locations, such as community pharmacies and outpatient clinics [20, 49, 50].

#### External factors

One of the most-cited barriers by both pharmacists and managers was the resistance to change of some of the pharmacists and health team, which can hinder the development of pharmaceutical clinical activities in the hospital. This barrier has also been reported in previous studies [51, 52]. According to Gastelurrutia et al. (2007), the resistance to change can be influenced by professionals’ fear to take on new responsibilities, lack of confidence, problems in education, conservatism, and the convenience of current practical activities [53].

According to the managers, the health team does not understand the clinical activities of pharmacists, this being another factor that contributes to resistance from both these professionals and other members of the health care team. In developing countries, the pharmacist does not yet have a prominent role in monitoring the use of medicines, illness prevention, and health promotion, being little recognized as a health professional by either society or the health care team [19, 48, 54]. According to Lin 50 years ago in the US, pharmacists were reluctant to change the focus of the profession from the production and delivery of drugs to services directed toward the patient [55]. This reluctance was justified by the comfort of carrying out traditional and consolidated tasks. However, as health care evolved, pharmacists’ responsibilities and expectations also changed, and these professionals did adapt to the new practice [56]. Thus, changes in attitude, effort, and persistence are necessary for achieving significant changes, at both personal and organizational levels, to meet these new demands and current social need [39, 57].

Flaws in the public health system that reverberate in hospital administration were highlighted by pharmacists as barriers that should be reduced to facilitate implementation of the services. The study by Marten et al. also reports that one of the barriers to the development of health services in developing countries are problems of the public system, and that the biggest challenge is the lack of political stability [58]. Issues such as financing, public-private partnership, and persistent inequalities cannot be resolved solely in the technical sphere. Thus, political organization and awareness is necessary for technical reforms to be truly effective [59]. Historical tensions between the health care team and managers of hospital institutions corroborate the hospital management problems cited by pharmacists. Conflicting priorities most often result in the will of directors being carried out, with cost-reducing measures used to justify budget cuts in materials for the hospital [60]. On the other hand, the health team may blame administrators for flaws that arise from non-compliance with their demands [61].

#### Structural and organizational

Barriers such poor hospital design were reported by pharmacists as difficult problems to solve, and are also cited in the study by Brazinha and Llimós (2014) [49]. However, for managers, structural difficulties of the hospital can be overcome through organization of processes and recognition by the hospital board of the positive outcomes after the implementation of clinical pharmacy services. According to Gutierrez (2012), the structure of an institution is one of the cornerstones underlying the quality of health services [62]. However, poor service is not only related to the lack of access to resources; greater investment does not guarantee efficiency or quality. Organizational changes are needed to optimize the use of available resources and thus improve the quality of health services [63].

Lack of time for clinical activities and a shortage of pharmacists for these services were reported by pharmacists and managers, which may be due to demands of administrative tasks. This is a reflection on the inadequate attribution of services for pharmacy assistants, barriers also considered in other studies [20, 64]. According to Doucette et al. (2012), it is possible to change the responsibilities and activities of the pharmacy assistants, which could increase efficiency and decrease the time commitment of the pharmacist to technical functions [50]. By freeing pharmacists from technical functions, new pharmacy services may be implemented.

#### Attitudes of pharmacists and relationship with the health team

In this present study, pharmacists reported feelings of shame, fear, and frustration owing to lack of knowledge and clinical skills, and that they were inadequately prepared by their pharmacy education. Lack of training for clinical activities is one of the factors reported in many studies about barriers to implementation of pharmaceutical clinical services [19, 20, 40]. In some countries, the education of pharmacists still focuses on the preparation of drugs to carry out industrial or laboratory science-centered activities, pushing this profession away from clinical activities [8, 9, 65]. In this context, the literature has advocated strategic investment in clinical training to bridge the gap in clinical skills. This would allow pharmacists acquire more confidence and certainty in relation to the provision of new pharmaceutical practices [20, 50].

Communication difficulties between the health care team were highlighted in the present study, as in other similar studies carried out in countries implementing clinical pharmacy services [19]. Factors such as lack of knowledge and communication skills, fear of invasion of other professionals’ space, as well as fear of other health professionals interfering in their work may hinder the pharmacist’s relationship with the team [48]. According to Berger (2011), the main reason for pharmacists contacting other health team members is when something is wrong or when a problem needs to be solved [66]. Thus, because conversations often begin with a problem, they can be seen in a negative or confrontational way, triggering defensive behavior in the professionals, thus hindering communication. However, effective communication between pharmacists and other health professionals can reduce medication errors in the hospital environment [67].

### Implementation facilitators

Pharmacists reported that one of the facilitators of implementation of the services is the high level of clinical experience of some pharmacists in the institution. According to the literature, previous experience of pharmacists was a significant factor in the implementation of outpatient clinical services in Australia, Brazil, Spain, and Finland [68–70].

Clinical pharmacy is in the consolidation phase in developing countries and few hospitals have professionals who have experienced this service [19]. According to Therrien (1997) and Perez & Maia (2011), articulating experiential knowledge (implicit, tacit) as well as theory, aids clinical practice in the working environment, and contributes to understanding of the services by inexperienced professionals [71]. Furthermore, frequent discussions and meetings can assist in the exchange of experiences [72].

Another implementation facilitator reported by managers was the participation of the pharmacist in multidisciplinary activities of the hospital. Strategies to promote collaboration include promoting effective communication, trust, and respect among pharmacists and other team professionals, as well as attempting to change the attitudes of the professionals [73].

Another point stressed was the importance of both maintaining records and disclosing positive clinical and economic impacts of the clinical pharmacy services implemented. According to the literature, the disclosure of positive results is a strategic marketing activity to demonstrate to the directors of the institution, other health professionals, and users the effectiveness of implemented services [74]. It provides clinical, humanistic, and economic evidence for expanding services for the entire hospital by capturing the awareness of the institution’s administrators and other health team members.

## Conclusion

In this study, participants identified workers’ resistance, fear, and frustration due to lack of clinical and communication skills as barriers. Besides that, poor facility layout has been identified as a difficult problem to solve.

The main expectations were related to the sustainability of clinical services, and development of clinical and communication skills. Moreover, the experience in clinical pharmacy of some pharmacists in vthe institution was one of the facilitators most cited by pharmacists. Moreover, the managers’ expectations included the participation of the pharmacist in multidisciplinary activities of the hospital, and the recording and disclosure of the clinical and economic impact of implemented services.

This study has identified factors that may influence the implementation of clinical pharmacy services in the institution analyzed. Thus, these results may be used in planning the implementation of clinical pharmacy in hospitals.

## Abbreviations

CFF: Federal council of pharmacy
COREQ: Consolidated criteria for reporting qualitative research
HUSE: Emergency hospital of sergipe
SUS: Unified health system
WHO: World Health Organization

## References

[1] Perez A, Touchette DR, Doloresco F, Hoffman JM, Meek PD, Touchette DR (2009). Economic evaluations of clinical pharmacy services: 2001– 2005. Pharmacotherapy.

[2] Rivkin A, Hongjun Y (2011). Evaluation of the role of the critical care pharmacist in identifying and avoiding or minimizing significant drug-drug interactions in medical intensive care patients. J Crit Care.

[3] Long E, Hu M, Tong R, Liu J (2014). Pharmacoeconomics evaluation of clinical pharmacy service for diabetic inpatients. Value Health.

[4] Ibrahim MM, Suh HS. Chapter 3 - Economic Evaluation of Pharmacy Services: Review of Studies From Asia, Africa, and South America. In Economic Evaluation of Pharmacy Services. 2017:35–97.

[5] Ghani K, Gillani W, Ghani M (2010). Pharmacy teaching and practices problems in developing countries: review. Int J Pharm Teach Pract.

[6] Pande S, Hiller JE, Nkansah N, Bero L. The effect of pharmacist-provided non-dispensing services on patient outcomes, health service utilization and costs in low-and middle-income countries. Cochrane Libr. 2013;28(2) 10.1002/14651858.CD010398 PMC9829534 23450614

[7] Smith F (2009). The quality of private pharmacy services in low and middle-income countries: a systematic review. Pharm World Sci.

[8] Allemann SS, Van Mil JF, Boterman L, Berger K, Griese N, Hersberger KE (2014). Pharmaceutical care: the PCNE definition 2013. Int J Clin Pharm.

[9] Khan TM, Ahmed KM, ANWAR MA (2011). Perspective for clinical pharmacy curriculum development and validation in Asian developing nations. J Young Pharm.

[10] Van Mil JF, Schulz M, Tromp TFD (2014). Pharmaceutical care, European developments in concepts, implementation, teaching, and research: a review. Pharm World Sci.

[11] ABENFAR (2010). Associação Brasileira de Ensino Farmacêutico.

[12] Brasil. Ministério da Saúde. (2008). Secretaria de Ciência, Tecnologia e Insumos Estratégicos. Departamento de Assistência Farmacêutica e Insumos Estratégicos I Fórum Nacional de Educação Farmacêutica: o farmacêutico de que o Brasil necessita: relatório final / Ministério da Saúde, Secretaria de Ciência, Tecnologia e Insumos Estratégicos, Departamento de Assistência Farmacêutica e Insumos Estratégicos. – Brasília: Editora do Ministério da Saúde, 68.

[13] Castro MS, Correr CJ (2007). Pharmaceutical care in community pharmacies: practice and research in Brazil. Ann Pharmacother.

[14] Cabana MD, Rand CS, Powe NR, Wu AW, Wilson MH, Abbound PAC, Rubin HR (1999). Why don't physicians follow clinical practice guidelines?: a framework for improvement. JAMA.

[15] Cochrane LJ, Olson CA, Murray S, Dupuis M, Tooman T, Hayes S (2007). Gaps between knowing and doing: understanding and assessing the barriers to optimal health care. J Contin Educ Health Prof.

[16] Erdogan ON, Erdogan MS, Gunay O, Erkus S, Ullus T (2012). Community pharmacists’ perception of their clinical pharmacy service function, a study from Turkey. Revista Farmacia.

[17] Kaae S, Christensen ST (2012). Exploring long term implementation of cognitive services in community pharmacies - a qualitative study. Pharm Pract.

[18] Sarriff A, Gillani WS, Abdel G, Babiker RM (2010). Pharmacist perception to importance and self-competence in pharmacy practice. Int J Pharm Stud Res.

[19] Penm J, Moles R, Wang H, Li Y, Chaar B (2014). Factors affecting the implementation of clinical pharmacy services in China. Qual Health Res.

[20] Dosea SA, Brito GC, Santos LMC, Marques TC, Balisa-Rocha B, Pimentel D, Bueno D, Lyra-Jr DP. Establishment, implementation, and consolidation of clinical pharmacy services in community pharmacies: perceptions of a group of pharmacists. Qual Health Res. 2015;27(3):363–73. 10.1177/1049732315614294 26658232

[21] Gil MI, Benrimoj SI, Martíner-Martinez F, Cardero M, Gastestelurrutia MÁ (2015). Priorization of facilitators for the implementation of medication review with follow-up service in Spanish community pharmacies through exploratory factor analysis. Atención Primaria.

[22] Minayo MCS. O desafio do conhecimento: pesquisa qualitativa em saúde. 13ª ed. São Paulo: Hucitec, Abrasco; 2013.

[23] Holloway I, Wheeler S. Qualitative research in nursing and healthcare3^rd^ edition: Wiley; 2013.

[24] Tong A, Sainsbury P, Craig J (2007). Consolidated criteria for reporting qualitative research (COREQ): a 32-item checklist for interviews and focus groups. Int J Qual Health Care.

[25] Bardin L. Análise de conteúdo. (1977). Lisboa (Portugal): Edições 3, vol. 70; 2010.

[26] Blazejewski L, Vaidya V, Pinto S, Gaither C (2013). Pharmacists’ perceived barriers providing non-dispensing services to underserved populations. J Community Health.

[27] Wibowo Y, Parsons R, Sunderland B, Hughes J (2015). Evaluation of community pharmacy-based services for type-2 diabetes in an Indonesian setting: pharmacist survey. Int J Clin Pharm.

[28] Bermond MD, Fernandes Z, Costa E, Cunha N, Honda A (2008). Modelo referencial de ensino para uma formação farmacêutica com qualidade.

[29] Simão BT, Oliveira L, Toreti Becker IR. A formação do profissional Farmacêutico e sua inserção na Atenção Básica. Revista do Programa de Residência Multiprofissional em Atenção Básica/Saúde da Família. 2013;1(1)

[30] Awalom MT, Tesfa AF, Kidane ME, Ghebremedhin MR, Teklesenbet AH (2015). Eritrean pharmacists’ job satisfaction and their attitude to re-professionalize pharmacy in to pharmaceutical care. Int J Clin Pharm.

[31] Kumanov IK (2016). The challenging paradigm of pharmaceutical care. Scripta Scientifica Pharmaceutica.

[32] Urbonas G, Kubilienė L. Assessing the relationship between pharmacists’ job satisfaction and over-the-counter counselling at community pharmacies. Int J Clin Pharm. 2016;38(2):252–60. 10.1007/s11096-015-0232-y 26666908

[33] Kreling DH, Doucette WR, Mott DA, Gaither CA, Pedersen CA, Schommer JC (2005). Community pharmacists’ work environments: evidence from the 2004 National Pharmacist Workforce Study. J Am Pharm Assoc: JAPhA.

[34] Brasil. Ministério da Saúde (2013). Portaria GM/MS n° 529, de 1 de abril de 2013. Institui o Programa Nacional de Segurança do Paciente (PNSP). Diário Oficial da União.

[35] Marchon SG, Mendes-Junior WV (2014). Segurança do paciente na atenção primária à saúde: revisão sistemática Patient safety in primary health care: a systematic review La seguridad del paciente en la atención primaria. Cad Saúde Pública.

[36] Brandão E, Oliveira P, Melo PFADS, Silva PDS, Felisberto N, Araújo J (2015). Implantação de um programa de segurança do paciente em uma unidade ambulatorial especializada em filariose linfática. Revista Acreditação.

[37] Organização Mundial de Saúde (OMS). (2012). APPS web-based registration mechanism open. Geneva; [Acesso em 2015 out 27]. Disponível em: http://www.who.int/patientsafety/en/

[38] Capucho HC, Cassiani SHDB (2013). Necessidade de implantar programa nacional de segurança do paciente no Brasil. Rev Saude Publica.

[39] Feletto E, Wilson LK, Roberts AS, Benrimoj SI (2010). Flexibility in community pharmacy: a qualitative study of business models and cognitive services. Pharm World Sci.

[40] Mansoor SM, Aslani P, Krass I (2014). Pharmacists’ attitudes and perceived barriers to provision of adherence support in Australia. Int J Clin Pharm.

[41] Roberts AS, Benrimoj SI, Chen TF, Williams KA, Aslani P (2008). Practice change in community pharmacy: quantification of facilitators. Ann Pharmacother.

[42] Woods P, Gapp R, King MA (2015). Researching pharmacist managerial capability: philosophical perspectives and paradigms of inquiry. Res Soc Adm Pharm.

[43] Andrade TNG, Silvestre CC, Cunha LC, Silva DT, Marques TC, Oliveira-Filho AD (2015). Pharmaceutical intervention assessment in the identification and management of drug interactions in an intensive care unit. J Appl Pharm Sci.

[44] Chung N, Rascati K, Lopez D, Jokerst J, Garza A (2014). Impact of a clinical pharmacy program on changes in hemoglobin a1c, diabetes-related hospitalizations, and diabetes-related emergency department visits for patients with diabetes in an underserved population. J Manage Care Spec Pharm.

[45] Gallagher PF, O'connor MN, O'mahony D (2011). Prevention of potentially inappropriate prescribing for elderly patients: a randomized controlled trial using STOPP/START criteria. Clin Pharmacol Ther.

[46] Magedanz L, Silliprandi EM, Santos RP (2012). Impact of the pharmacist on a multidisciplinary team in an antimicrobial stewardship program: a quasi-experimental study. Int J Clin Pharm.

[47] Colla CH, Mainor AJ, Hargreaves C, Sequist T, Morden N. Interventions aimed at reducing use of low-value health services: a systematic review. Med Care Res Rev. 2017;74(5):507–50. 10.1177/1077558716656970 27402662

[48] Reis TM, Guidoni CM, Girotto E, Rascado RR, Carvalho MP, Cruciol JM (2015). Pharmaceutical care in Brazilian community pharmacies: knowledge and practice. Afr J Pharm Pharmacol.

[49] Bhagavathula AS, Sarkar BR, Patel I (2014). Clinical pharmacy practice in developing countries: focus on India and Pakistan. Arch Pharm Pract.

[50] Brazinha I, Fernandez-Llimos F (2014). Barriers to the implementation of advanced clinical pharmacy services at Portuguese hospitals. Int J Clin Pharm.

[51] Doucette WR, Nevins JC, Gaither C, Kreling DH, Mott DA, Pedersen CA (2012). Organizational factors influencing pharmacy practice change. Res Soc Adm Pharm.

[52] Al Khalidi D, Wazaify M (2013). Assessment of pharmacists’ job satisfaction and job related stress in Amman. Int J Clin Pharm.

[53] Zardaín E, Del Valle MO, Loza MI, García E, Lana A, Markham WA, López ML (2009). Psychosocial and behavioural determinants of the implementation of Pharmaceutical Care in Spain. Pharm World Sci.

[54] Gastelurrutia MA, Fernandez-Llimos F, Benrimoj SI, Castrillon CC, Faus MJ (2007). Barriers for the implementation of cognitive services in Spanish community pharmacies. Aten Primaria.

[55] OPAS - Organização Pan-Americana de Saúde (2002). Consenso Brasileiro de Atenção Farmacêutica: proposta.

[56] Lin YY (2012). Evolution of pharm D education and patient service in the USA. J Exp Clin Med.

[57] Abramowitz PW (2009). The evolution and metamorphosis of the pharmacy practice model. Harvey a.K. Whitney. Am J Health Syst Pharm.

[58] Roberts AS, Benrimoj SC, Chen TF, Williams KA, Hopp TR, Aslani P (2005). Understanding practice change in community pharmacy: a qualitative study in Australia. Res Soc Adm Pharm.

[59] Marten R, Mcintyre D, Travassos C, Shishkin S, Longde W, Reddy S, Vega J (2014). An assessment of progress towards universal health coverage in Brazil, Russia, India, China, and South Africa (BRICS). Lancet.

[60] Paim J, Travassos C, Almeida C, Bahia L, Macinko J (2011). Saúde no Brasil 1 O sistema de saúde brasileiro: história, avanços e desafios. Lancet.

[61] Vaghetti HH, Padilha MICS, LunardiFilho WD, Lunardi VL, Costa CD (2011). Significados das hierarquias no trabalho em hospitais públicos brasileiros a partir de estudos empíricos. Acta Paulista de Enfermagem.

[62] Merry, M.D. (2005). Hospital-medical staff culture clash: is it inevitable or preventable? Health Trustees of New York State. The challenge of Governance. [Acesso em 2015 out 26]. Disponível em: http://scholar.googleusercontent.com/scholar?q=cache:1NEAVfD3o1EJ:scholar.google.com/&hl=pt-BR&as_sdt=0,5.

[63] Gutierrez JP. The structural quality of health services as a potential constraint for human capital accumulation: Health related behaviours as determinants of wellbeing, Forthcoming; 2012. Available at SSRN: https://ssrn.com/abstract=1982040.

[64] Kunkel S, Rosenqvist U, Westerling R (2007). The structure of quality systems is important to the process and outcome, an empirical study of 386 hospital departments in Sweden. BMC Health Serv Res.

[65] Hipólito E (2015). Comportamento dos farmacêuticos, indicadores de estrutura e processos das farmácias comunitárias privadas do Estado do Paraná. (2015). [dissertação].

[66] Gastelurrutia MA (2005). Elementos facilitadores y dificultades para la diseminación e implantación de servicios cognitivos del farmacêutico en la farmácia comunitária española. (2005). [tesis].

[67] Berger BA. In: de Lyra Junior TDP, et al., editors. Habilidades de comunicação para farmacêuticos: construindo relacionamentos, otimizando o cuidado aos pacientes. 3rd ed. São Paulo: Pharmabooks; 2011.

[68] Rixon S, Braaf S, Williams A, Liew D, Manias E. Pharmacists’ interprofessional communication about medications in specialty hospital settings. Health Commun. 2015;30(11):1065–75. 10.1080/10410236.2014.919697 25317781

[69] Alcaraz JC, Mas RP, Sanchís MC, Mongars AG, García EG, Ferrer FB (2015). Valoración de una Encuesta sobre Servicios de Atención Farmacéutica a Farmacéuticos de Alicante. Pharmaceutical Care España.

[70] Benrimoj RA, Chen SI, Williams T, Gadiel MD, Ridoutt ML. An investigation into business and professional facilitators for change for the pharmacy profession in light of the Third Guild/Government Agreement Sydney: University of Sydney; 2003.

[71] Leikola S. Development and Application of Comprehensive Medication Review Procedure to Community – Dwelling Elderly, Doctoral Dissertation, University of Helsinki; 2012. Available at: https://helda.helsinki.fi/bitstream/handle/10138/30203/developm.pdf?sequence%BC1

[72] Therrien J (1997). Saber de experiência, identidade e competência profissional: Como os docentes produzem sua profissão. Contexto Educação.

[73] Rocha G, Russi A, Alvarez J (2013). Etnoeducação patrimonial–reflexões antropológicas em torno de uma experiência de formação de professores. Revista Pro-posições.

[74] Snyder ME, Zillich AJ, Primack BA, Rice KR, Mcgivney MAS, Pringle JL, Smith RB (2010). Exploring successful community pharmacist-physician collaborative working relationships using mixed methods. Res Soc Adm Pharm.

